# One‐year persistent symptoms and functional impairment in SARS‐CoV‐2 positive and negative individuals

**DOI:** 10.1111/joim.13482

**Published:** 2022-03-31

**Authors:** Mayssam Nehme, Olivia Braillard, François Chappuis, Delphine S. Courvoisier, Laurent Kaiser, Paola M. Soccal, Jean‐Luc Reny, Frederic Assal, Guido Bondolfi, Aglaé Tardin, Christophe Graf, Dina Zekry, Silvia Stringhini, Hervé Spechbach, Frederique Jacquerioz, Julien Salamun, Frederic Lador, Matteo Coen, Thomas Agoritsas, Lamyae Benzakour, Riccardo Favale, Stéphane Genevay, Kim Lauper, Philippe Meyer, Nana K. Poku, Basile N. Landis, Stéphanie Baggio, Marwène Grira, José Sandoval, Julien Ehrsam, Simon Regard, Camille Genecand, Garance Kopp, Ivan Guerreiro, Gilles Allali, Pauline Vetter, Idris Guessous, CoviCare Study Team

**Affiliations:** ^1^ Division of Primary Care Medicine Geneva University Hospitals Geneva Switzerland; ^2^ Division of Tropical and Humanitarian Medicine Geneva University Hospitals Geneva Switzerland; ^3^ Faculty of Medicine University of Geneva Geneva Switzerland; ^4^ Cantonal Health Service General Directorate for Health Geneva Switzerland; ^5^ Quality of Care Division Medical Directorate Geneva University Hospitals Geneva Switzerland; ^6^ Division of Infectious Diseases Geneva University Hospitals Geneva Switzerland; ^7^ Geneva Center for Emerging Viral Diseases Geneva University Hospitals Geneva Switzerland; ^8^ Division of Laboratory Medicine Laboratory of Virology Geneva University Hospitals Geneva Switzerland; ^9^ Division of Pulmonary Medicine Geneva University Hospitals Geneva Switzerland; ^10^ Division of General Internal Medicine Geneva University Hospitals Geneva Switzerland; ^11^ Division of Neurology Geneva University Hospitals Geneva Switzerland; ^12^ Division of Psychiatry Geneva University Hospitals Geneva Switzerland; ^13^ Department of Rehabilitation and Geriatrics Geneva University Hospitals Geneva Switzerland; ^14^ Division of Rheumatology Geneva University Hospitals Geneva Switzerland; ^15^ Division of Cardiology Geneva University Hospitals Geneva Switzerland; ^16^ Division of Otolaryngology Geneva University Hospitals Geneva Switzerland; ^17^ Division of Prison Health Geneva University Hospitals Geneva Switzerland; ^18^ Department of Oncology Geneva University Hospitals Geneva Switzerland; ^19^ Department of Medical Information Sciences Geneva University Hospitals Geneva Switzerland; ^20^ Division of Emergency Medicine Geneva University Hospitals Geneva Switzerland

**Keywords:** epidemiology, functional impairment, infectious diseases, inflammation, internal medicine, persistent symptoms, post‐COVID, SARS

## Abstract

**Background:**

Persistent symptoms of SARS‐CoV‐2 are prevalent weeks to months following the infection. To date, it is difficult to disentangle the direct from the indirect effects of SARS‐CoV‐2, including lockdown, social, and economic factors.

**Objective:**

The study aims to characterize the prevalence of symptoms, functional capacity, and quality of life at 12 months in outpatient symptomatic individuals tested positive for SARS‐CoV‐2 compared to individuals tested negative.

**Methods:**

From 23 April to 27 July 2021, outpatient symptomatic individuals tested for SARS‐CoV‐2 at the Geneva University Hospitals were followed up 12 months after their test date.

**Results:**

At 12 months, out of the 1447 participants (mean age 45.2 years, 61.2% women), 33.4% reported residual mild to moderate symptoms following SARS‐CoV‐2 infection compared to 6.5% in the control group. Symptoms included fatigue (16% vs. 3.1%), dyspnea (8.9% vs. 1.1%), headache (9.8% vs. 1.7%), insomnia (8.9% vs. 2.7%), and difficulty concentrating (7.4% vs. 2.5%). When compared to the control group, 30.5% of SARS‐CoV‐2 positive individuals reported functional impairment at 12 months versus 6.6%. SARS‐CoV‐2 infection was associated with the persistence of symptoms (adjusted odds ratio [aOR] 4.1; 2.60–6.83) and functional impairment (aOR 3.54; 2.16–5.80) overall, and in subgroups of women, men, individuals younger than 40 years, those between 40–59 years, and in individuals with no past medical or psychiatric history.

**Conclusion:**

SARS‐CoV‐2 infection leads to persistent symptoms over several months, including in young healthy individuals, in addition to the pandemic effects, and potentially more than other common respiratory infections. Symptoms impact functional capacity up to 12 months post infection.

## Graphical abstract

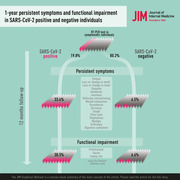

## Introduction

Post‐acute sequelae of SARS‐CoV‐2 infection (PASC), long COVID, or post‐COVID syndrome [1, 2, 3] have now been shown to be prevalent to varying degrees following the infection. Prevalence varies between 10% and 30% [4, 5, 6, 7] in the first few months following the infection, and up to 70% [8] depending on the study population. Taking lessons from history and past infections [9], symptoms may persist for months to years in some patients, as already described in conditions following the severe acute respiratory syndrome (SARS) outbreak in 2003 [10], the Middle East respiratory syndrome (MERS) in 2012 [11], and other viruses such as the Epstein–Barr virus [12], or bacteria such as *Coxiella burnetii* [12]. To date, few studies have assessed the prevalence of symptoms at 12 months after SARS‐CoV‐2 infection and most were limited to post‐hospitalized settings only [13, 14, 15]. The long‐term prevalence of symptoms in outpatient settings has been described at several weeks to months post infection [5, 6, 16] but the information is still missing on the chronicity and prolonged evolution of these symptoms, as well as the burden of disease.

Additionally, only a few studies using patient records have compared post SARS‐CoV‐2 symptoms to the general population of adults tested negative for the infection [17, 18, 19], potentially shedding light on the direct versus indirect effects of the virus due to lockdown and pandemic‐related measures. A recent study assessing PASC among seropositive versus seronegative healthcare workers showed a higher prevalence of symptoms lasting at least 2 months in the seropositive group, with disruption of their work, social, and home life [20]. Determining the consequences due to the virus in addition to those due to postponed healthcare [21], socioeconomic disruption [22], or the mental health burden of the pandemic [23] is extremely important. This has led many in the scientific community to call on studies comparing sequelae post SARS‐CoV‐2 infection with control groups, beyond information from patient records only [9]. Control groups have been difficult to establish as the available testing (reverse transcriptase polymerase chain reaction [RT‐PCR] or antigenic testing in the acute phase, serology in the later phases) could lead to false negatives owing in part to the time of testing, access to test, and immune response [24].

In this study, we describe the prevalence of symptoms, functional capacity, productivity, and quality of life at 12 months in individuals tested positive for SARS‐CoV‐2 compared to individuals tested negative during the same time period (defined as the control group). The study hypothesis postulates that SARS‐CoV‐2 positive individuals suffer more from persistent symptoms leading to functional impairment on top of the pandemic effects when compared to SARS‐CoV‐2 negative individuals. We also assess the association between SARS‐CoV‐2 infection and the long‐term persistence of symptoms.

## Methods

From 23 April to 27 July 2021, an online questionnaire was sent to all adults, 12 months after being tested for SARS‐CoV‐2 infection (RT‐PCR test between 15 March 2020 and 30 July 2020) at the outpatient testing center of the Geneva University Hospitals (Switzerland). Overall, 3515 adults had a valid mobile phone number or email address, and were contacted for follow‐up. Individuals tested positive for SARS‐CoV‐2 were considered as the group of interest for SARS‐CoV‐2 infection and those tested negative for SARS‐CoV‐2 were considered as the control group. All individuals gave consent and the study was approved by the Cantonal Research Ethics Commission of Geneva, Switzerland (protocol number 2021‐00389). Only individuals with a laboratory‐confirmed test date were included in this study.

The questionnaire was distributed via REDCap, and a personal link was sent to each participant via email or SMS in case an email address was not available. The follow‐up questionnaire included questions about baseline characteristics, comorbidities, self‐rated health, symptoms at the time of testing, evolution of symptoms since testing, current symptoms over the past 2 weeks, symptom intensity and frequency when present, treatment, number of contacts with the healthcare system (hospitalizations and visits to the primary care physician or other specialists), functional capacity, productivity, and quality of life.

Age categories were defined as “below 40,” “40–59 years,” and “60 years and above” on the basis of previous studies suggesting that middle age may be a predictor of persistent symptoms [16]. Symptoms at testing were categorized as asymptomatic (self‐reported “no symptoms”), paucisymptomatic (self‐reported “very few symptoms” at testing), or symptomatic (self‐reported “had several symptoms” at testing). Symptoms’ intensity in the 2 weeks preceding the questionnaire was assessed using validated scales when possible—the Chalder fatigue scale [25] and the Eastern Cooperative Oncology Group (ECOG) performance scale [26] for fatigue, the modified Medical Research Council scale for dyspnea [27], the Insomnia Severity Index for sleeping disorders [28], the Hospital Anxiety and Depression (HAD) scale for psychiatric conditions [29], and a Likert scale with self‐reported options of mild, moderate, or severe for all reported symptoms. The bimodal scoring of the Chalder fatigue scale [25] was used and scores were rated as 0–3 (no fatigue) and 4 or more (fatigue present). Symptoms’ frequency in the 2 weeks preceding the questionnaire was assessed using a Likert scale with self‐reported options of never, rarely, often, or always. Quality of life was assessed using the 12‐item Short Form survey (SF‐12) questionnaire [30], and physical component score (PCS) and mental component score (MCS) were calculated based on the answers; these scores generally have means of 50 and standard deviations (SDs) of 10 in the general US population [30]. A score of 50 or less on the PCS can be used to indicate a physical condition, and a score of 42 or less on the MCS can be used to indicate potential clinical depression [31, 32]. Functional capacity was assessed using the Sheehan Disability Scale [33], assessing functional impairment in three domains—professional, social, and family life—using a 10‐point visual scale with 0 (no impairment at all), 1–3 (mild impairment), 4–6 (moderate impairment), 7–9 (marked impairment), and 10 (extreme impairment), as well as numerical values for days lost and days with reduced productivity due to functional impairment [33] in the week preceding the survey. Self‐rated health was assessed using the first question of the SF‐12 questionnaire [30] consisting of a Likert scale of excellent, very good, good, fair, and poor; answers were later categorized into two categories: 0 (poor to fair) and 1 (good to excellent). See Supporting Information for the complete survey instrument.

Data were collected using REDCap v11.0.3 and analyzed using the statistical software Stata, version 16.0 (StataCorp). Descriptive analyses included percentages with comparisons using chi‐square tests or Fisher's exact test when appropriate. A *p*‐value <0.05 was considered significant. Prevalence estimates of each symptom were adjusted for the time from infection, age, sex, education, profession, working in healthcare setting, smoking, physical activity, COVID‐19 vaccination status, symptoms at presentation, hospitalization, and the following comorbidities when present prior to testing: overweight or obese, hypertension, respiratory disease, cardiovascular disease, diabetes, immunosuppression, hypothyroidism, anemia, migraine, tension headache, sleeping disorder, anxiety, depression, any psychiatric condition, irritable bowel syndrome, chronic pain syndrome, and chronic fatigue.

Logistic regression models were used to evaluate associations between SARS‐CoV‐2 infection and persistent symptoms at 12 months, as well as SARS‐CoV‐2 infection and functional impairment at 12 months. Only participants reporting onset of new symptoms at testing were included in the regression analysis, in order to ensure that symptoms were new in nature. The outcome of persistence of symptoms at 12 months was defined as having at least one symptom in the past 2 weeks preceding the questionnaire. The outcome of functional impairment was defined as having mild, moderate, or severe functional impairment using the Sheehan Disability Scale [33]. Multivariable regression models were used to calculate adjusted odds ratios (aORs) with a 95% confidence interval. aORs were adjusted for time from infection, age, sex, education, profession, working in healthcare setting, smoking, physical activity, COVID‐19 vaccination status, symptoms at presentation, hospitalization, and the following comorbidities when present prior to testing: overweight or obese, hypertension, respiratory disease, cardiovascular disease, diabetes, immunosuppression, hypothyroidism, anemia, migraine, tension headache, sleeping disorder, anxiety, depression, any psychiatric condition, irritable bowel syndrome, chronic pain syndrome, and chronic fatigue. Additional analyses were conducted to determine the association between SARS‐CoV‐2 infection and the persistence of symptoms or functional impairment at 12 months across subgroups of sex, age, and individuals without any past medical or psychiatric history. The subgroup analysis for individuals without any past medical or psychiatric history aimed to mitigate any potential bias from underlying conditions that could lead to similar persistent symptoms independent of SARS‐CoV‐2 infection.

## Results

**Fig. 1:**
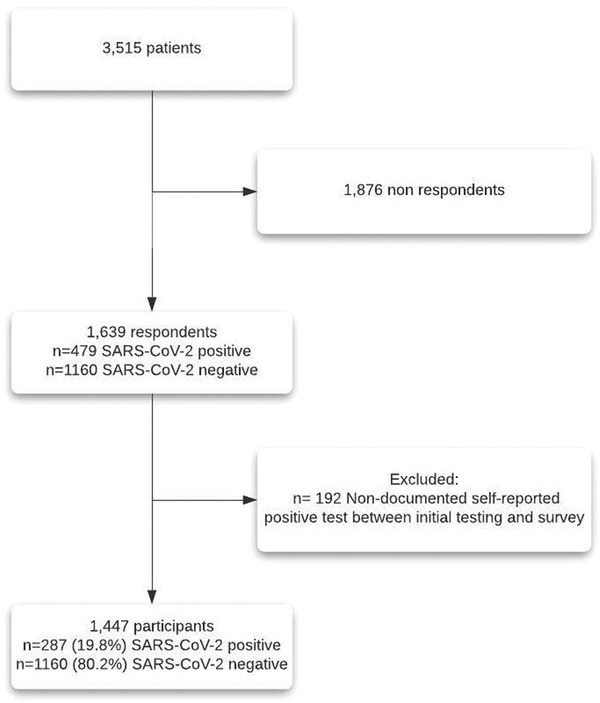
Flowchart.

Overall, 3515 persons received the questionnaire—*n* = 703 (20%) with a positive test result and 2812 (80%) with a negative test result. Of those invited to participate, 1639 persons answered the questionnaire (46.6% response rate), with a mean age of 45.4 years (SD, 14.6), 60.8% were women, and 29.2% had a positive SARS‐CoV‐2 test, compared to nonresponders, who had a mean age of 47.8 years (SD, 15.9) (*p* < 0.001) and of whom 56.2% were women (*p* < 0.001). Of the 1639 persons answering the questionnaire, 192 reported a positive test between the initial test and the follow‐up and were excluded because of lack of a laboratory‐confirmed test date and result. Ultimately, 1447 persons were included in the analysis (Fig. 1).

The mean age of the included participants was 45.2 years (SD, 14.5) and 61.2% were women. When considering baseline characteristics (Table 1), 50.5% of participants had never smoked and 41.8% did not have any comorbidities prior to the infection. Of the 1447 participants, *n* = 287 (19.8%) had a laboratory‐confirmed SARS‐CoV‐2 infection and *n* = 1160 (80.2%) had a negative RT‐PCR test, *n* = 194 (13.4%) self‐reported hospitalization since their test date, with *n* = 33 (11.5%) reporting their hospitalization was due to SARS‐CoV‐2 infection. The median time from testing to survey was 367 days (interquartile range [IQR], 349–378) overall, 388 days (IQR, 372–399) for individuals who tested positive, and 364 days (IQR, 346–374) for individuals who tested negative.

**Table 1:** Overall characteristics of participants tested positive and negative for SARS‐CoV‐2

		SARS‐CoV‐2	SARS‐CoV‐2	
	Total	Positive	Negative	
	(*n* = 1447)	(*n* = 287)	(*n* = 1160)	*p*‐Value
Age ± SD (years)	45.2 ± 14.5	44.2 ± 13.2	45.5 ± 14.8	0.179
Characteristic	*N* (%)	*N* (%)	*N* (%)	
Age categories				0.001
Below 40	599 (41.4)	115 (40.1)	484 (41.7)	
40–59 years	589 (40.7)	139 (48.4)	450 (38.8)	
60 years and above	259 (17.9)	33 (11.5)	226 (19.5)	
Sex				0.160
Male	561 (38.8)	101 (35.2)	460 (39.7)	
Female	886 (61.2)	186 (64.8)	700 (60.3)	
Employment status				0.009
Salaried	997 (68.9)	220 (76.7)	777 (67.0)	
Retired	131 (9.0)	12 (4.2)	119 (10.3)	
Student	118 (8.1)	20 (7.0)	98 (8.5)	
Independent	76 (5.3)	13 (4.5)	63 (5.4)	
Looking after home/family	39 (2.7)	6 (2.1)	33 (2.8)	
Unemployed	39 (2.7)	6 (2.1)	33 (2.8)	
Disability	27 (1.9)	3 (1.0)	24 (2.1)	
Other	20 (1.4)	7 (2.4)	13 (1.1)	
Education				0.017
Primary	72 (5.0)	21 (7.3)	51 (4.4)	
Apprenticeship	165 (11.4)	23 (8.0)	142 (12.2)	
Secondary	202 (14.0)	46 (16.0)	156 (13.5)	
Tertiary	902 (62.3)	170 (59.2)	732 (63.1)	
Other	75 (5.2)	22 (7.7)	53 (4.6)	
Prefer not to answer	31 (2.1)	5 (1.7)	26 (2.2)	
Profession				<0.001
Never worked	91 (6.3)	21 (7.3)	70 (6.0)	
Unskilled workers	94 (6.5)	10 (3.5)	84 (7.2)	
Skilled workers	256 (17.7)	39 (13.6)	217 (18.7)	
Highly skilled workers	338 (23.4)	103 (35.9)	235 (20.3)	
Professional—managers	415 (28.7)	59 (20.6)	356 (30.7)	
Other	226 (15.6)	52 (18.1)	174 (15.0)	
Prefer not to answer	27 (1.9)	3 (1.0)	24 (2.1)	
Smoking status				<0.001
Never smoked	731 (50.5)	162 (56.4)	569 (49.0)	
Current smoker	296 (20.5)	32 (11.1)	264 (22.8)	
Previous smoker, stopped independent of infection	378 (26.1)	78 (27.2)	300 (25.9)	
Previous smoker, stopped due to infection	2 (0.1)	2 (0.7)	0 (0.0)	
Prefer not to answer	40 (2.8)	13 (4.5)	27 (2.3)	
Physical activity				0.785
None	236 (16.3)	46 (16)	190 (16.4)	
Partial	710 (49.1)	141 (49.1)	569 (49.1)	
Regular	489 (33.8)	99 (34.5)	390 (33.6)	
Prefer not to answer	12 (0.8)	1 (0.3)	11 (0.9)	
Hospitalization since test	194 (13.4)	33 (11.5)	161 (13.9)	0.262
Vaccination				<0.001
No	458 (31.6)	145 (50.5)	313 (27.0)	
2 doses	714 (49.4)	84 (29.3)	630 (54.3)	
1 dose	262 (18.1)	54 (18.8)	208 (17.9)	
Prefer not to answer	13 (0.9)	4 (1.4)	9 (0.8)	
Symptoms at presentation				<0.001
Asymptomatic	331 (22.9)	22 (7.7)	309 (26.7)	
Paucisymptomatic	691 (47.7)	221 (77.0)	470 (40.5)	
Had several symptoms	419 (29.0)	44 (15.3)	375 (32.3)	
Prefer not to answer	6 (0.4)	0 (0.0)	6 (0.5)	
Comorbidities				
None	605 (41.8)	132 (46.0)	473 (40.8)	0.109
Overweight	213 (14.7)	41 (14.3)	172 (14.8)	0.817
Sleep disorders	178 (12.3)	31 (10.8)	147 (12.7)	0.388
Hypertension	157 (10.9)	25 (8.7)	132 (11.4)	0.193
Anxiety	127 (8.8)	21 (7.3)	106 (9.1)	0.329
Migraine	123 (8.5)	25 (8.7)	98 (8.4)	0.886
Irritable bowel syndrome	99 (6.8)	14 (4.9)	85 (7.3)	0.141
Chronic fatigue syndrome	91 (6.3)	20 (7.0)	71 (6.1)	0.596
Depression	89 (6.2)	16 (5.6)	73 (6.3)	0.650
Respiratory disease	88 (6.1)	15 (5.2)	73 (6.3)	0.498
Tension headache	62 (4.3)	10 (3.5)	52 (4.5)	0.455
Anemia	61 (4.2)	11 (3.8)	50 (4.3)	0.718
Memory disorders	58 (4.0)	19 (6.6)	39 (3.4)	0.012
Tendinitis	57 (3.9)	9 (3.1)	48 (4.1)	0.435
Obesity	55 (3.8)	15 (5.2)	40 (3.4)	0.158
Cardiovascular disease	54 (3.7)	10 (3.5)	44 (3.8)	0.805
Attention disorders	44 (3.0)	12 (4.2)	32 (2.8)	0.209
Diabetes	43 (3.0)	9 (3.1)	34 (2.9)	0.855
Hypothyroidism	43 (3.0)	7 (2.4)	36 (3.1)	0.553
Immunosuppression	29 (2.0)	1 (0.3)	28 (2.4)	0.025
Cancer	20 (1.4)	3 (1.0)	17 (1.5)	0.585
Chronic pain syndrome	20 (1.4)	4 (1.4)	16 (1.4)	0.985
Deep vein thrombosis	19 (1.3)	3 (1.0)	16 (1.4)	0.656
Hyperthyroidism	15 (1.0)	2 (0.7)	13 (1.1)	0.526
Rheumatoid arthritis	11 (0.8)	3 (1.0)	8 (0.7)	0.535
Multiple sclerosis	10 (0.7)	0 (0.0)	10 (0.9)	0.114
Renal disease	9 (0.6)	0 (0.0)	9 (0.8)	0.134
Fibromyalgia	9 (0.6)	1 (0.3)	8 (0.7)	0.510
Dysmenorrhea	8 (0.6)	1 (0.3)	7 (0.6)	0.602
Reactive arthritis	8 (0.6)	2 (0.7)	6 (0.5)	0.713

*Note*: SARS‐CoV‐2 status was determined as per the result of the reverse transcriptase polymerase chain reaction test in symptomatic outpatient individuals.

Abbreviation: SD, standard deviation.

At 12 months, an estimated 33.4% of individuals post SARS‐CoV‐2 infection still had at least one symptom compared to 6.5% in the control group (*p* < 0.001). Significant differences according to SARS‐CoV‐2 infection were seen in the prevalence of fatigue and several neurologic, psychiatric, upper respiratory, and cardiac symptoms (Table 2). The number of symptoms at 12 months was increased following a documented SARS‐CoV‐2 infection with 7.8% of individuals with SARS‐CoV‐2 infection reporting 5–10 symptoms at 12 months compared to 1.1% of individuals in the control group (*p* < 0.001). Table 2 shows the adjusted prevalence of each symptom at 12 months following testing.

**Table 2:** Prevalence and number of symptoms in individuals who tested positive and individuals who tested negative for SARS‐CoV‐2 at 12 months^a^, ^b^

	SARS‐CoV‐2 positive	SARS‐CoV‐2 negative	
	(*n* = 287)	(*n* = 1160)	
Symptom	% (95% CI)	% (95% CI)	*p*‐Value
Any symptom	33.4 (31.3–35.5)	6.5 (6.1–6.8)	<0.001
Fatigue	16.0 (14.4–17.5)	3.1 (2.7–3.3)	<0.001
Loss or change in smell	10.0 (8.6–11.5)	1.7 (1.6–1.9)	<0.001
Loss or change in taste	10.3 (7.8–12.8)	1.5 (1.2–1.9)	<0.001
Dyspnea	8.9 (6.6–11.2)	1.1 (0.9–1.3)	<0.001
Headache	9.8 (8.4–11.2)	1.7 (1.5–2.0)	<0.001
Insomnia	8.9 (5.4–12.3)	2.7 (1.6–3.9)	<0.001
Difficulty concentrating/loss of memory	7.4 (5.8–9.1)	2.5 (2.0–3.0)	<0.001
Mental exhaustion	6.9 (5.0–8.8)	1.4 (1.1–1.7)	<0.001
Paresthesia	5.4 (3.1–7.8)	1.9 (1.5–2.3)	<0.001
Dizziness/lack of equilibrium	6.4 (4.4–8.5)	1.8 (1.0–2.5)	<0.001
Cough	6.5 (3.4–9.7)	3.2 (1.8–4.6)	0.033
Chest pain	6.0 (2.9–9.2)	4.0 (2.3–5.7)	0.214
Palpitations	3.8 (1.6–6.1)	1.6 (0.6–2.5)	0.032
Myalgia	7.3 (6.2–8.4)	1.7 (1.5–1.9)	<0.001
Arthralgia	2.9 (2.0–3.9)	2.0 (1.7–2.4)	0.050
Digestive symptoms (nausea, vomiting, diarrhea, abdominal pain)	4.0 (3.0–4.9)	1.6 (1.2–2.0)	<0.001
Number of symptoms			
None	67.8 (65.8–69.7)	93.6 (93.2–93.9)	<0.001
1 symptom	10.6 (10.1–11.1)	2.7 (2.6–2.9)	<0.001
2 symptoms	4.8 (4.5–5.0)	1.0 (0.9–1.1)	<0.001
3 symptoms	5.0 (4.7–5.3)	0.9 (0.8–1.0)	<0.001
4 symptoms	2.8 (2.6–3.0)	0.5 (0.4–0.5)	<0.001
5–10 symptoms	7.8 (7.2–8.5)	1.1 (1.1–1.2)	<0.001
≥11 symptoms	1.1 (1.0–1.2)	0.1 (0.1–0.1)	<0.001

Abbreviation: CI, confidence interval.

^a^ “Any symptom” was defined as the presence of any one symptom of those listed in the survey instrument.

^b^ Estimates of prevalence were adjusted for time from infection, age, sex, education, profession, working in healthcare setting, smoking, physical activity, COVID‐19 vaccination, symptoms at presentation, hospitalization, and the following comorbidities present prior to testing: overweight or obese, hypertension, respiratory disease, cardiovascular disease, diabetes, immunosuppression, hypothyroidism, anemia, migraine, tension headache, sleeping disorder, anxiety, depression, any psychiatric condition, irritable bowel syndrome, chronic pain syndrome, and chronic fatigue.

Functional impairment was more important in individuals who tested positive compared to those tested negative for SARS‐CoV‐2, and so were the numbers of days lost and days with reduced productivity in the week preceding the follow‐up (Table 3. After adjustment, an estimated 30.5% of individuals who tested positive reported functional impairment at 12 months from the infection versus 6.6% of individuals who tested negative (*p* < 0.001). In the week preceding the questionnaire, participants reported to have experienced one or more days of reduced productivity in the week preceding the questionnaire in 11.8% of cases compared to 3.9% in the control group (*p* < 0.001).

**Table 3:** Functional impairment of individuals who tested positive compared to individuals who tested negative for SARS‐CoV‐2 at 12 months using the Sheehan Disability Scale^a^ [33]

		SARS‐CoV‐2	SARS‐CoV‐2	
	Total	Positive	Negative	
	(*n* = 1447)	(*n* = 287)	(*n* = 1160)	
	% (95% CI)	% (95% CI)	% (95% CI)	*p*‐Value
Functional impairment				
No impairment	88.8 (88.1–89.5)	69.5 (67.5–71.7)	93.4 (93.1–93.8)	<0.001
Mild impairment	5.9 (5.6–6.2)	14.5 (13.7–15.3)	3.8 (3.6–4.0)	<0.001
Moderate impairment	4.2 (3.9–4.5)	12.2 (11.2–13.2)	2.2 (2.1–2.4)	<0.001
Severe impairment	1.1 (1.0–1.2)	3.6 (3.2–4.0)	0.5 (0.4–0.6)	<0.001
≥1 day lost in the past week	2.8 (2.2–3.3)	4.8 (3.4–6.2)	2.3 (1.7–2.8)	0.002
≥1 day of reduced productivity in the past week	5.5 (5.0–6.1)	11.8 (10.1–13.5)	3.9 (3.4–4.4)	<0.001

Abbreviation: CI, confidence interval.

^a^ Estimates were adjusted for time from infection, age, sex, education, profession, working in healthcare setting, smoking, physical activity, COVID‐19 vaccination status, symptoms at presentation, hospitalization, and the following comorbidities present prior to testing: overweight or obese, hypertension, respiratory disease, cardiovascular disease, diabetes, immunosuppression, hypothyroidism, anemia, migraine, tension headache, sleeping disorder, anxiety, depression, any psychiatric condition, irritable bowel syndrome, chronic pain syndrome, and chronic fatigue.

**Fig. 2:**
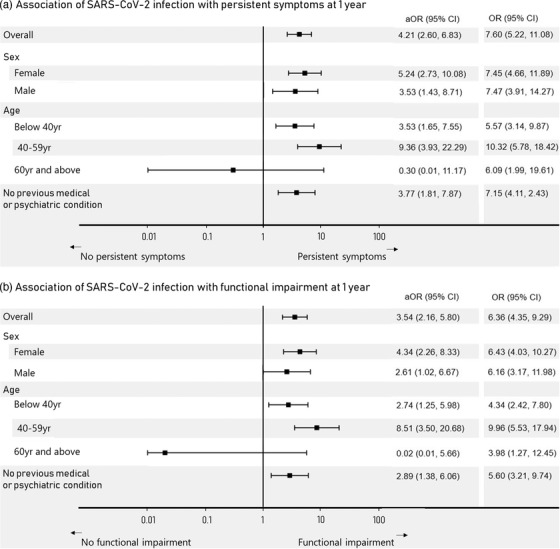
Associations between laboratory‐confirmed SARS‐CoV‐2 infection and the persistence of symptoms and functional impairment at 1 year stratified by sex, age categories, and pre‐existing medical conditions (n = 1245). The persistence of symptoms was defined as the presence of any one symptom within the 2 weeks before the questionnaire. Functional impairment was assessed using the Sheehan Disability Scale [33]. Odds ratios were adjusted for time from infection, age, sex, education, profession, working in healthcare setting, smoking, physical activity, COVID‐19 vaccination status, symptoms at presentation, hospitalization, and the following comorbidities present prior to testing: overweight or obese, hypertension, respiratory disease, cardiovascular disease, diabetes, immunosuppression, hypothyroidism, anemia, migraine, tension headache, sleeping disorder, anxiety, depression, any psychiatric condition, irritable bowel syndrome, chronic pain syndrome, and chronic fatigue. aOR, adjusted odds ratio; CI, confidence interval; OR, odds ratio.

SARS‐CoV‐2 infection was positively associated with the persistence of symptoms at 12 months (aOR 4.21; 2.60–6.83). The association was significant in the subgroups of women, men, individuals younger than 40 years, and those between 40–59 years of age. The association was also significant for individuals without any past medical or psychiatric conditions (Fig. 2). SARS‐CoV‐2 infection was positively associated with functional impairment at 12 months (aOR 3.54; 2.16–5.80). The association was significant in the subgroups of women, men, in individuals younger than 40 years, and in those between 40–59 years of age. The association was also significant for individuals without any past medical or psychiatric conditions (Fig. 2).

Self‐rated health was reported as very good to excellent in 47.4% of individuals at 12 months post SARS‐CoV‐2 infection, as good in 38.8% of individuals, and as fair to poor in 13.8% of individuals (Table 4). The mean weighted physical health score using the SF‐12 questionnaire was 49.7 (SD, 7.9) for individuals post SARS‐CoV‐2 infection compared to 50.9 (SD, 7.8) for the control group (*p* = 0.017). The mean weighted mental health score using the SF‐12 questionnaire was 41.8 (SD, 5.5) for individuals post SARS‐CoV‐2 infection compared to 41.0 (SD, 5.9) for the control group (*p* = 0.041). Additionally, the mean HAD anxiety score was 5.5 (SD, 3.9) for individuals post SARS‐CoV‐2 infection compared to 6.2 (SD, 3.9) for the control group (*p* = 0.008). The mean HAD depression score was 3.4 (SD, 3.6) for individuals post SARS‐CoV‐2 infection versus 3.7 (SD, 3.5) for the control group (*p* = 0.189). Further details are presented in Table 4.

**Table 4:** Quality of life and physical and mental health of individuals who tested positive compared to individuals who tested negative for SARS‐CoV‐2 at 12 months^a^, ^b^

		SARS‐CoV‐2	SARS‐CoV‐2	
	Total	Positive	Negative	
	(*n* = 1447)	(*n* = 287)	(*n* = 1160)	
Quality of life—SF12	% (95% CI)	% (95% CI)	% (95% CI)	*p*‐Value
Current self‐rated health				
Excellent	17.8 (17.0–18.6)	14.4 (12.9–15.8)	18.6 (17.7–19.5)	<0.001
Very good	34.8 (34.1–35.4)	33.0 (31.3–34.6)	35.2 (34.5–35.9)	0.007
Good	36.0 (35.2–36.8)	38.8 (37.2–40.5)	35.4 (34.5–36.3)	<0.001
Fair	10.4 (9.8–11.0)	12.7 (11.1–14.2)	9.9 (9.2–10.5)	<0.001
Poor	0.9 (0.8–1.1)	1.1 (0.9–1.4)	0.9 (0.7–1.0)	0.096
SF‐12 scores				
SF‐12 physical component score (mean ± SD)	50.7 ± 7.8	49.7 ± 7.9	50.9 ± 7.8	0.017
SF‐12 mental component score (mean ± SD)	41.2 ± 5.9	41.8 ± 5.4	41.0 ± 5.9	0.041
HAD—Anxiety and Depression	% (95% CI)	% (95% CI)	% (95% CI)	
HAD Anxiety score ≥8	29.0 (27.9–30.0)	21.9 (19.7–24.1)	30.7 (29.5–31.9)	<0.001
HAD Depression score ≥8	12.8 (12.1–13.5)	9.9 (8.6–11.3)	13.4 (12.6–14.3)	0.001
HAD Anxiety score ≥11	11.3 (10.6–12.0)	8.3 (6.9–9.6)	12.0 (11.2–12.9)	<0.001
HAD Depression score ≥11	4.2 (3.7–4.8)	4.6 (3.4–5.7)	4.2 (3.6–4.7)	0.561
HAD scores				
HAD Anxiety score (mean ± SD)	6.1 ± 3.9	5.5 ± 3.9	6.2 ± 3.9	0.008
HAD Depression score (mean ± SD)	3.7 ± 3.5	3.4 ± 3.6	3.7 ± 3.5	0.189

*Note*: A score of more than 8 on each of the anxiety or depression components of HAD^29^ indicates a positive score.

Abbreviations: CI, confidence interval; HAD, Hospital Anxiety and Depression scale; SD, standard deviation; SF‐12, Short Form 12 item.

^a^ In SF‐12, a physical component score of 50 or less indicates a physical condition, while a mental component score of 42 may be indicative of clinical depression.^30^

^b^ Estimates were adjusted for time from infection, age, sex, education, profession, working in healthcare setting, smoking, physical activity, COVID‐19 vaccination status, symptoms at presentation, hospitalization, and the following comorbidities present prior to testing: overweight or obese, hypertension, respiratory disease, cardiovascular disease, diabetes, immunosuppression, hypothyroidism, anemia, migraine, tension headache, sleeping disorder, anxiety, depression, any psychiatric condition, irritable bowel syndrome, chronic pain syndrome, and chronic fatigue.

## Discussion

At 12 months, persistent symptoms were more prevalent in individuals who tested positive compared to individuals who tested negative for SARS‐CoV‐2 after adjusting for sociodemographic variables and comorbidities. The increased prevalence is significant for most symptoms with the largest differential seen for fatigue, headache, dyspnea, insomnia, myalgia, difficulty concentrating, and loss/change in taste or smell. SARS‐CoV‐2 infection led to significantly more functional impairment when compared to the control group, as well as up to three times less productivity at 12 months from testing. Increased functional impairment has been suggested in seropositive healthcare workers [20] and in smaller studies including post‐hospitalized patients [34]. Our results show functional impairment in outpatient individuals post SARS‐CoV‐2 infection. The overall PCS on the SF‐12 scale was lower in the SARS‐CoV‐2 positive group compared to the SARS‐CoV‐2 negative group, indicating potentially poorer physical health. The overall MCS was low in both groups, indicating an overall impact on quality of life. The mental score component and the anxiety and depression scale scores were lower in the group of individuals without SARS‐CoV‐2 infection compared to individuals post SARS‐CoV‐2 infection, possibly due to underlying baseline characteristics where the control group reported more anxiety and depression than the SARS‐CoV‐2 group [35]. One hypothesis is that people with more anxiety or depression might have opted to stay home or be less socially active during the pandemic, thus decreasing their risk of SARS‐CoV‐2 infection [36, 37].

The prevalence of persistent symptoms is in line with several recent studies and compares to infections like SARS in 2003 [10] and MERS [11], where symptoms lingered and caused functional impairment for years in a significant proportion of individuals. SARS‐CoV‐2 may, however, cause a higher clinical burden of disease than other respiratory viruses, as suggested in recent studies comparing long‐term sequelae post hospitalization for SARS‐CoV‐2 versus influenza [17, 38, 39]. As influenza is also known to cause potential declines in functional capacity, especially in hospitalized patients [40], a comparison of the functional impairment following SARS‐CoV‐2 infection versus influenza would bring further insight into the overall burden of disease. Mechanisms leading to long‐term symptoms of SARS‐CoV‐2 are still being studied, as well as a potential treatment and preventive approaches [41]. In a recent study, vaccination was associated with a decreased prevalence of symptoms at 28 days post infection [42], potentially due to the fact that vaccinated individuals experienced less symptomatic infections [42].

The importance of this study is showing the differential impact of SARS‐CoV‐2 infection versus other potential upper respiratory infections in symptomatic outpatient individuals, and in addition to the potential general pandemic toll. Additionally, our study shows that symptoms can persist at least 12 months after the infection (alpha strain of SARS‐CoV‐2) and are associated with more functional impairment and loss of productivity when compared to other common upper respiratory infections or pandemic‐related effects in general. Individuals suffering from PASC should be cared for long term, with a multidisciplinary approach addressing the range of their symptoms and any potential impact on their productivity and activities of daily living. These findings also underline the importance of continuing to follow public health recommendations that aim to reduce infection, regardless of age, sex, or underlying conditions in order to avoid persistent symptoms.

Limitations in this study include the nature of the follow‐up with self‐reported items as well as the relatively low response rate, which, although similar to other general survey questionnaires [43], may result in ascertainment bias. Additionally, several of the assessed symptoms, such as fatigue, loss of taste or smell, and others, are subjective and self‐reported in nature in clinical practice as well as in research settings. The time from infection was shorter for participants who tested positive compared to those who tested negative, potentially leading to bias, although with a difference of only 20 days. Time from infection was controlled for in all analyses. The age group of 60 years and older included a smaller number of participants and a low number of SARS‐CoV‐2 positive individuals, thus potentially leading to a lack of power in the multivariable regression models and loss of associations. Additionally, we considered a negative RT‐PCR test as negative for a SARS‐CoV‐2 infection. While it is possible for individuals with a negative RT‐PCR test to still have SARS‐CoV‐2 infection [44], the decision to include only laboratory‐confirmed infection, potentially biasing results towards the null hypothesis, was made to objectively assess the duration of symptoms as narrowly as possible as well as the impact of SARS‐CoV‐2 infection versus other potential factors. Finally, more women participated in the study, and participants were highly educated, skilled workers, raising the issue of generalizability of the results to the general population. Of note, profession and education were controlled for in all analyses, and all participants were tested at the same testing center and had similar access to care, thus mitigating this factor as a potential confounder leading to symptoms’ persistence.

To conclude, SARS‐CoV‐2 infection causes persistent symptoms at 12 months from diagnosis in addition to the COVID‐19 pandemic effects, and more than other potential upper respiratory infections. As the COVID‐19 landscape continues to evolve with new treatment options, vaccination, and new variants, physicians should continue monitoring their patients and encouraging them to avoid infection and re‐infection regardless of age, sex, and underlying conditions in order to reduce the risk of PASC.

## Conflict of interest

The authors declare no potential conflict of interest. Furthermore, we confirm that the manuscript has been read and approved by all the named authors, and that there are no other persons who satisfied the criteria for authorship but are not listed. We further confirm that this work was conducted with the ethical approval of the Cantonal Research Ethics Commission of Geneva, Switzerland, and that the approvals are acknowledged in the manuscript.

## Author contributions

Mayssam Nehme: Conceptualization; formal analysis; investigation; methodology; writing – original draft; writing – review and editing. Olivia Braillard: Conceptualization; data curation; writing – review and editing. François Chappuis: Conceptualization; writing – original draft; writing – review and editing. Laurent Kaiser: Data curation; writing – review and editing. Idris Guessous: Conceptualization; data curation; funding acquisition; methodology; project administration; resources; supervision; writing – original draft; writing – review and editin. All authors of the CoviCare Study team contributed to the study design and reviewing the manuscript.

## Funding

This study is funded by the Leenaards Foundation, the Geneva University Hospitals Private Foundation, and the Private Research Funds of the Division of Primary Care Medicine at the Geneva University Hospitals. The funders of the study had no role in the study design, data collection, data analysis, data interpretation, or writing of the manuscript.

## Supporting information




**Supplement 1**. Survey instrument

## Data Availability

Individual study data that underlie the results reported in this article can be made available to the scientific community after de‐identification and upon submission of a data request application to the investigator board via the corresponding author.
