# Supplementary material for: One‐year persistent symptoms and functional impairment in SARS‐CoV‐2 positive and negative individuals
Source: J Intern Med. 2022 Mar 31;292(1):103–15. doi: 10.1111/joim.13482 (PMC9115262; doi:10.1111/joim.13482)
Supplement: Supplementary file 1 — Supplement 1. Survey instrument [file JOIM-292-103-s001.docx]

| **Supplement 1. Survey instrument** | | |  | |
| --- | --- | --- | --- | --- |
| Employment status | Salaried  Retired  Student  Independent  Looking after home/family  Unemployed  Disability  Other | | | |
|  |  |  |  |  |
|  |  |  |  |  |
|  |  |  |  |  |
|  |  |  |  |  |
|  |  |  |  |  |
|  |  |  |  |  |
|  |  |  |  |  |
| Education | Primary (compulsory education, no formal education)  Apprenticeship  Secondary (secondary education, specialized schools)  Tertiary (universities, higher professional education, doctorates)  Other  Prefer not to answer | | | |
|  |  |  |  |  |
|  |  |  |  |  |
|  |  |  |  |  |
|  |  |  |  |  |
|  |  |  |  |  |
|  |  |  |  |  |
| Profession | Unskilled workers (manual labour, craftsmen, traders, farmers, employees without specific training)  Skilled workers (employees with specific training)  Highly skilled workers (employees with intermediate training)  Professional-Managers (company managers with more than 10 employees, individuals with a profession requiring university training)  Independent (consultants, were independent or were company managers with fewer than 10 employees)  Other  Prefer not to answer | | | |
|  |  |  |  |  |
|  |  |  |  |  |
|  |  |  |  |  |
|  |  |  |  |  |
| Work in healthcare setting | Yes (Hospital, clinic, nursing home, homecare)  No | | | |
| Smoking status | I have never smoked  I am a current smoker  I am an ex-smoker, but stopped prior to my SARS-CoV-2 infection  I am an ex-smoker, but stopped after my SARS-CoV-2 infection | | | |
| Physical activity | I do not do any physical activity  I am partially active  I am completely active | | | |
| Compared to before the test date, your physical activity is: | The same  Less  More | | | |
| Do you suffer from any of the following conditions (multiple answers possible) | None  Overweight  Sleep disorders  Migraine  Hypertension  Anxiety  Irritable bowel syndrome  Depression  Chronic fatigue syndrome  Respiratory disease  Other arthritic disorder (specify)  Tension headache  Tendinitis  Obesity  Anaemia  Attention disorders  Memory disorders  Hypothyroidism  Other digestive disorder (specify)  Cardiovascular disease  Other type of headache (specify)  Diabetes  Chronic pain syndrome  Immunosuppression  Deep vein thrombosis  Dysmenorrhea  Hyperthyroidism  Rheumatoid arthritis  Other neurologic disorder (specify)  Fibromyalgia  Ulcerative colitis  Multiple sclerosis  Cancer  HIV  Other psychiatric disorder (specify)  Reactive arthritis  Renal disease  Ankylosing spondylitis  Crohn disease  Lupus  Sjogren disease  Cirrhosis | | | |
| If condition checked, specify | [This condition] is new since my test date  I suffer from [this condition] since before my test date  Prefer not to answer | | | |
| Did you have COVID-19 compatible symptoms at time of testing? | Yes, I had symptoms  Yes, but very few symptoms  No, I did not have symptoms  Prefer not to answer | | | |
| After your test date, how did your symptoms evolve? | I never had symptoms  My symptoms disappeared  My symptoms are fluctuating  My symptoms are constant  Other  Prefer not to answer | | | |
| What was or has been the duration of symptoms since they started? | 0-10 days  11-20 days  21-30 days  1-2 months  2-3 months  3-4 months  4-5 months  5-6 months  6-7 months  8-9 months  9-10 months  10-11 months  11-12 months  More than 12 months  Do not know | | | |
| After your laboratory confirmed test at the Geneva University Hospitals, did you have any nasopharyngeal swab (RT-PCR or antigenic test) positive for COVID-19? | Yes  No | | If yes, date of test | |
| Have you had any serological testing for COVID-19 | Yes  No | | If yes, date of test and result | |
| Have you been vaccinated against SARS-CoV-2 | Yes, I received 1 dose  Yes, I received 2 doses  No  Prefer not to answer | | If yes, date of each dose | |
| Which type of vaccine did you receive | Comirnaty® (BNT162b2) vaccine of Pfizer/BioNTech  (mRNA-1273) vaccine of Moderna  Vaxzevria (previously COVID-19 vaccine of Oxford/AstraZeneca)  Sinopharm BIBP vaccine of China National Pharmaceutical Group  Sputnik V vaccine of Gamaleya Research Institute of Epidemiology and Microbiology  Janssen Vaccine of Johnson&Johnson  Do not know  Prefer not to answer  Other | | | |
| Did you receive any of the following treatments in the first 21 days after your test date? (multiple answers possible) | None  Paracetamol  Non-steroidal anti-inflammatory medication  Antiviral (ritonavir, lopinavir, remdesivir etc.)  Dexamethasone, prednisone or other steroid  Monoclonal antibodies (Tocilizumab etc.)  Inhaled spray (Seretide®, Ventolin®, Symbicort®, Spiriva®, Atrovent®, Bricanyl®, Dospir®, etc.)  Nasal spray (Nasonex®, Rhinomer® etc.)  Hydroxychloroquine (Plaquenil®)  Ivermectin  Anticoagulation (Lovenox®, Arixtra® etc.)  Homeopathic treatment  Zinc  Vitamin C  Vitamin D  Other  Prefer not to answer | | | |
| Have you been hospitalized since your test date? | Yes  No  Prefer not to answer | | If yes, was the hospitalization related to COVID-19 and date of hospitalization | |
| Have you seen a physician or healthcare professional in relation to your symptoms? (multiple answers possible) | Yes, my primary care physician  Yes, I have been to the emergency room  Yes, I have seen another specialist, physician or healthcare professional (specify)  No | | If yes, how many times for each answer and is this a new follow-up since your test date? | |
| In the past 2 weeks, which of the following symptoms have you experienced, even if fluctuating? (multiple answers possible) | Fatigue  Headache  Change in smell  Mental exhaustion  Myalgia  Dyspnoea  Difficulty concentrating  Insomnia  Stress  Change in taste  Loss of memory  Paraesthesia  Arthralgia  Neck pain  Anxiety  Cough  Sadness  Loss of smell  Palpitations  Dizziness  Back pain  Throat pain  Diarrhoea  Nausea  Abdominal pain  Loss of taste  Hair loss  Lack of equilibrium  Lack of appetite  Chest pain  Constipation  Generalized pain  Fever > 38 C  Rash  Malaise  Vomiting  Toe pain or redness  Finger pain or redness  Other  Prefer not to answer | | | |
| If symptom checked, did you have this symptom regularly (most days) prior to the test? | Yes  No  Prefer not to answer | | | |
| Current symptom intensity | Mild  Moderate  Severe | | | |
| Current symptom frequency | Never  Rarely  Often  Always | | | |
| If fatigue, specify | Normal activity, no limitations in daily activity  Limited activity but capable to do light work (office work, cleaning)  Limited activity but <50% in bed during the day  >50% in bed, but not bedbound  Bedbound | | | |
| If fatigue, | Do you have problems with tiredness? | | Yes  No | |
|  | Do you need to rest more? | | Yes  No | |
|  | Do you feel sleepy or drowsy? | | Yes  No | |
|  | Do you have problems starting things? | | Yes  No | |
|  | Do you lack energy? | | Yes  No | |
|  | Do you have less strength in your muscles? | | Yes  No | |
|  | Do you feel weak? | | Yes  No | |
|  | Do you have difficulty concentrating? | | Yes  No | |
|  | Do you make slips of the tongue when speaking? | | Yes  No | |
|  | Do you find it more difficult to find the right word? | | Yes  No | |
|  | Is your memory as good as usual? | | Yes  No | |
| If dyspnoea, specify | Dyspnoea only with strenuous exercise  Dyspnoea when hurrying or walking up a slight hill  Has to stop for breath when walking at own pace on flat surface  Stops for breath after walking 90 meters or after a few minutes  Too breathless to leave house or breathless when dressing | | |  |
| If insomnia, specify (over the past 2 weeks) | Do you have difficulty falling asleep? | | None  Mild  Moderate  Severe  Very severe | |
|  | Do you have difficulty staying asleep? | | None  Mild  Moderate  Severe  Very severe | |
|  | Do you have problems waking up too early? | | None  Mild  Moderate  Severe  Very severe | |
|  | How satisfied/dissatisfied are you with your current sleep pattern? | | Very satisfied  Satisfied  Moderately satisfied  Dissatisfied  Very dissatisfied | |
|  | How noticeable to others do you think your sleep problem is in terms of impairing the quality of your life? | | Not at all noticeable  A little  Somewhat noticeable  Very much noticeable | |
|  | How worried/distressed are you about your current sleep problem? | | Not at all worried  A little  Somewhat worried  Very much worried | |
|  | To what extent do you consider your sleep problem to interfere with your daily functioning currently? | | Not at all interfering  A little  Somewhat interfering  Very much interfering | |
| If symptom checked, specify | Your symptoms have disrupted your work/school work | | Scale:  0 Not at all – 10 Extremely | |
|  | Your symptoms have disrupted your social life/leisure activities | | Scale:  0 Not at all – 10 Extremely | |
|  | Your symptoms have disrupted your family life/home responsibilities | | Scale:  0 Not at all – 10 Extremely | |
|  | On how many days in the last week did your symptoms cause you to miss school or work or leave you unable to carry out your normal daily responsibilities? | | | |
|  | On how many days in the last week did you feel so impaired by your symptoms that even though you went to school or work, your productivity was reduced? | | | |
| In general, would you say your health prior to the test date was | Excellent, very good, good, fair, poor | |  | |
| In general, would you say your health is currently | Excellent, very good, good, fair, poor | |  | |
| The following questions are about activities you might do during a typical day. Does your health now limit you in these activities? If so, how much? | Moderate activities such as moving a table, pushing a vacuum cleaner, bowling, or playing golf | | Yes, limited a lot  Yes, limited a little  No, not limited at all | |
|  | Climbing several flights of stairs | | Yes, limited a lot  Yes, limited a little  No, not limited at all | |
| During the past 4 weeks, have you had any of the following problems with your work or other regular daily activities as a result of your physical health? | Accomplished less than you would like | | Yes  No | |
|  | Were limited in the kind of work or other activities | | Yes  No | |
| During the past 4 weeks, have you had any of the following problems with your work or other regular daily activities as a result of any emotional problems (such as feeling depressed or anxious)? | Accomplished less than you would like | | Yes  No | |
|  | Did work or activities less carefully than usual | | Yes  No | |
| During the past 4 weeks, how much did pain interfere with your normal work (including work outside the home and housework)? | | | Not at all  A little bit  Moderately  Quite a bit  Extremely | |
| These questions are about how you have been feeling during the past 4 weeks.  For each question, please give the one answer that comes closest to the way you have been feeling. How much of the time during the past 4 weeks… | Have you felt calm and peaceful? | | All of the time  Most of the time  A good bit of the time  Some of the time  A little of the time  None of the time | |
|  | Did you have a lot of energy? | | All of the time  Most of the time  A good bit of the time  Some of the time  A little of the time  None of the time | |
|  | Have you felt down-hearted and blue? | | All of the time  Most of the time  A good bit of the time  Some of the time  A little of the time  None of the time | |
| During the past 4 weeks, how much of the time has your physical health or emotional problems interfered with your social activities (like visiting friends, relatives, etc.)? | | | All of the time  Most of the time  A good bit of the time  Some of the time  A little of the time  None of the time | |
| I feel tense or wound up | |  | Most of the time  A lot of the time  From time to time, occasionally  Not at all | |
| I still enjoy the things I used to enjoy | |  | Definitely as much  Not quite so much  Only a little  Hardly at all | |
| I get a sort of frightened feeling as if something awful is about to happen | |  | Very definitely  Yes, but not too badly  A little, but it does not worry me  Not at all | |
| I can laugh and see the funny side of things | |  | As much as I always could  Not quite so much now  Definitely not so much now  Not at all | |
| Worrying thoughts go through my mind | |  | A great deal of the time  A lot of the time  From time to time, but not too often  Only occasionally | |
| I feel cheerful | |  | Not at all  Not often  Sometimes  Most of the time | |
| I can sit at ease and feel relaxed | |  | Definitely  Usually  Not often  Not at all | |
| I feel as if I am slowed down | |  | Nearly all the time  Very often  Sometimes  Not at all | |
| I get a sort of frightened feeling like 'butterflies' in the stomach | |  | Not at all  Occasionally  Quite often  Very often | |
| I have lost interest in my appearance | |  | Definitely  I do not take as much care as I should  I may not take quite as much care  I take just as much care as ever | |
| I feel restless as I have to be on the move | |  | Very much indeed  Quite a lot  Not very much  Not at all | |
| I look forward with enjoyment to things | |  | As much as I ever did  Rather less than I used to  Definitely less than I used to  Hardly at all | |
| I get sudden feelings of panic | |  | Very often indeed  Quite often  Not very often  Not at all | |
| I can enjoy a good book or radio or television program | |  | Often  Sometimes  Not often  Very seldom | |
| Have you ever been followed by a psychiatrist for any mental health condition prior to March 2020 (including depression, anxiety, sleeping disorders, suicidal ideations, fear of others, trauma, other problems) | |  | Yes  No | |
